# Preoperative accelerated radiotherapy combined with chemotherapy in a defined cohort of patients with high risk soft tissue sarcoma: a Scandinavian Sarcoma Group study

**DOI:** 10.1186/s13569-020-00145-5

**Published:** 2020-11-17

**Authors:** Kirsten Sundby Hall, Øyvind S. Bruland, Bodil Bjerkehagen, Elisabet Lidbrink, Nina Jebsen, Hans Hagberg, Karin Papworth, Oskar Hagberg, Clement Trovik, Henrik Bauer, Mikael Eriksson

**Affiliations:** 1 Department of Oncology, The Norwegian Radium Hospital, Oslo University Hospital, Oslo, Norway; 2 Institute for Clinical Medicine, Faculty of Medicine, University of Oslo, Oslo, Norway; 3 Department of Pathology, The Norwegian Radium Hospital, Oslo University Hospital, Oslo, Norway; 4 Institute for Oral Biology, Faculty of Dentistry, University of Oslo, Oslo, Norway; 5 Department of Medical Oncology, Karolinska University Hospital, Stockholm, Sweden; 6 Departments of Oncology and Orthopedics, Haukeland University Hospital and Centre for Cancer Biomarkers (CCBIO), University of Bergen, Bergen, Norway; 7 Department of Oncology, Uppsala University Hospital, Uppsala, Sweden; 8 Department of Oncology, Norrlands University Hospital, Umeå, Sweden; 9 Institution of Translation Medicine, Lund University, Malmö, Sweden; 10 Department Musculo-Skeletal Tumor Service/Orthopedics, Haukeland University Hospital, Bergen, Norway; 11 Musculo-Skeletal Tumor Service, Karolinska University Hospital, Stockholm, Sweden; 12 Department of Oncology, Skåne University Hospital, and Lund University, Lund, Sweden

**Keywords:** Soft tissue sarcoma, Preoperative, Adjuvant, Chemotherapy, Radiotherapy, Accelerated, Survival, Toxicity

## Abstract

**Background:**

We recently reported outcomes from a Scandinavian Sarcoma Group adjuvant study (SSG XX group A) conducted on localized and operable high risk soft tissue sarcoma (STS) of the extremities and trunk wall. SSG XX, group B, comprised of patients in a defined cohort with locally advanced STS considered at high risk for intralesional surgery. These patients received preoperative accelerated radiotherapy, together with neoadjuvant and adjuvant chemotherapy. Herein we report the results of this group B.

**Methods:**

Twenty patients with high-grade, locally advanced and deep STS located in lower extremities (n = 12), upper extremities (5) or trunk wall (3) were included. The median age was 59 years and 14 patients were males. The treatment regimen consisted of 6 cycles of doxorubicin (60 mg/m^2^) and ifosfamide (6 g/m^2^), with three cycles given neoadjuvantly, and preoperative radiotherapy (1, 8 Gyx2/daily to 36 Gy) between cycles 2 and 3. After a repeated MRI surgery was then conducted, and the remaining 3 chemotherapy cycles were given postoperatively at 3 weeks intervals. Survival data, local control, toxicity of chemotherapy and postoperative complications are presented.

**Results:**

Median follow-up time for metastasis-free survival (MFS) was 2.8 years (range 0.3–10.4). The 5-year MFS was 49.5% (95% confidence interval [CI] 31.7–77.4). The median follow-up time was 5.4 years (range 0.3–10.4) for overall survival (OS). The 5-year OS was 64.0% (95% CI 45.8–89.4). The median tumour size was 13 cm, with undifferentiated pleomorphic sarcoma (n = 10) and synovial sarcoma (n = 6) diagnosed most frequently. All patients completed surgery. Resection margins were R0 in 19 patients and R1 in 1 patient. No patients had evidence of disease progression preoperatively. Three patients experienced a local recurrence, in 2 after lung metastases had already been diagnosed. Eleven patients (55%) had postoperative wound problems (temporary in 8 and persistent in 3).

**Conclusions:**

Preoperative chemotherapy and radiotherapy were associated with temporary wound-healing problems. Survival outcomes, local control and toxicities were deemed satisfactory when considering the locally advanced sarcoma disease status at primary diagnosis.

*Trial registration* This study was registered at ClinicalTrials.gov Identifier NCT00790244 and with European Union Drug Regulating Authorities Clinical Trials No. EUDRACT 2007-001152-39

## Background

Preoperative chemotherapy (CT) and radiotherapy (RT) have previously been studied as an integrated treatment plan for patients with primary soft tissue sarcomas (STS) [1, 2]. Several potential benefits of preoperative chemo-/radiotherapy have been suggested, including an ability to assess primary tumour response to a given chemotherapeutic regimen and to facilitate radical surgical removal, as well as to initiate earlier systemic treatment to combat subclinical metastatic disease. In a recent study the Italian and Spanish Sarcoma Groups have shown, such combined treatment is feasible and safe with limited increase in wound complications [3].

According to formal guidelines [4], doxorubicin and ifosfamide are still not regarded as standard treatment in patients with high-grade localised soft-tissue sarcoma, neither as adjuvant nor neoadjuvant chemotherapy, despite a demonstrated benefit in several studies [5–10]. Our prospective, non-randomised Scandinavian Sarcoma Group (SSG) clinical study, SSG XX, was also designed to investigate the combination of adjuvant doxorubicin and ifosfamide in high risk STS [11]. In group A planned for primary surgery, accelerated radiotherapy was scheduled postoperatively between cycles 3 and 4 with a 5-year metastasis-free survival of 70.4% and overall survival 76.1% [11]. The increasing evidence for the benefit of adjuvant RT [12, 13] led to the introduction of an accelerated RT administered between courses of chemotherapy in SSG’s former protocol SSG XIII [14] and pursued also in SSG XX [11]. The SSG XX protocol had a separate treatment arm (group B) devoted to patients with locally advanced STS considered to have a high risk for intralesional surgery. These patients were given both pre- and postoperative chemotherapy, as well as accelerated RT before surgery. Here we report our experiences regarding the feasibility and outcomes of this combination.

## Methods

### Criteria for inclusion

The main eligibility criteria for SSG XX, group B were age ≥ 18 to ≤ 75 years, WHO performance status ≤ 1, and a locally advanced primary STS of high-grade histology (Grade III or IV in a 4 graded scale) located in the extremities or trunk wall. Only patients with a tumour of anatomical location and/or extension implying an obvious risk for intralesional margins were eligible. Tumour size was defined as the longest diameter on MRI at diagnosis. Tumour depth was defined in relation to the deep fascia. The patients were treated at four sarcoma centres in Sweden and two in Norway. Most cases were discussed at a surgical sarcoma network, a SSG web-based forum established to evaluate the risk for intralesional surgery and the need for preoperative treatment.

A core-needle biopsy or an open surgical biopsy was performed for classification of tumour type, grading of malignancy and histological diagnosis provided by the local pathologist. Diagnosis based on fine needle cytology alone was not accepted. The SSG Pathology Reference Group later reviewed the morphology in all cases according to the WHO-classification and malignancy grade, applying the Broders’ system [15–17]. Distinct grading may be unreliable in core-needle biopsies, and the pathologists considered the best assessment to be low grade and high grade, recognising that high grade will encompass both Broders’ grade 3 and 4. A core needle biopsy does not allow definition of the histological risk factors (vascular invasion, infiltrative growth, necrosis) as used in SSGXX, group A [11].

The following histiotypes were not eligible: extraskeletal osteosarcoma or chondrosarcoma, Ewing sarcoma, rhabdomyosarcoma, Kaposi sarcoma, clear cell sarcoma, alveolar soft part sarcoma, epithelioid sarcoma and radiation induced sarcoma.

Mandatory investigations at screening, during treatment and at follow-up visits have previously been presented [11]. The complete SSG XX protocol is available on the SSG website [18].

### Treatment

**Fig. 1:**
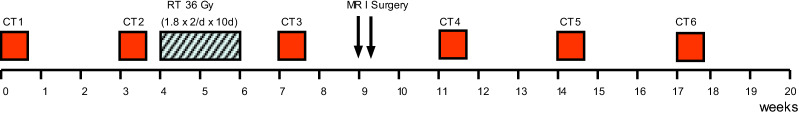
Treatment schedule. Chemotherapy (CT): ≥ 18 and < 70 years of age: Day 1: doxorubicin 60 mg/m^2^, 4-h infusion (IV); Day 1, 2 and 3: ifosfamide 2 g/m^2^/day as 2-h infusion, dose per cycle 6 g/m^2^ [with an equal dose of 2-mercaptoethane sulphonate sodium (MESNA)]. ≥ 70 and ≤ 75 years: doxorubicin 50 mg/m^2^ and ifosfamide 5 g/m^2^ (given as above). Granulocyte colony-stimulating factor was given routinely after each cycle

An outline of the treatment, involving scheduling of MRI, CT, RT and surgery, is presented in Fig. 1. A maximum of 28 days was allowed from diagnostic biopsy to start of chemotherapy. Doxorubicin 60 mg/m^2^ and ifosfamide 6 g/m^2^ were given with a 3 weeks interval for patients < 70 years of age and with doses of 50/5 from age ≥ 70 years. Details of CT and use of granulocyte colony-stimulating factor (G-CSF) have previously been published [11].

Accelerated RT allowing shortened treatment time was interposed from week 4 to 6, after completion of the two initial CT cycles and before the 3rd cycle (Fig. 1). This scheduling allowed the maintenance of a high overall dose intensity of the CT given. The fractionation schedule was 1.8 Gy twice daily to 36 Gy, with at least 6 h interval between the two daily fractions and 5 treatment days per week. Due to a radiosensitising effect of doxorubicin, the minimal interval between doxorubicin (cycle 2) and RT was set to 7 days. Clinical target volume (CTV) was defined by adding a 2 cm margin in all directions to the gross tumour volume (GTV), based on the baseline MRI examination. The choice of the conformal radiation treatment technique was decided by each centre.

Surgery was planned 3–4 weeks after RT to minimise the fibrotic tissue response. A preoperative MRI was repeated to document any changes in tumour size and extension. Two weeks after surgery, the first of the three remaining cycles of chemotherapy was given (Fig. 1).

All patients underwent surgery at a sarcoma centre. At that time, their neutrophil levels should be ≥ 1.0 × 10^9^/l and thrombocytes ≥  80 × 10^9^/l. The classification of margins, according to the SSG guidelines [18], was cooperatively determined by the surgeons and pathologists at each sarcoma center.

Details of RT for SSG XX groups A and B combined will be published later, with emphasis on quality assessment of the radiation dose distributions and target volume definitions in correlation with local recurrences, as well as formal scoring of late effects.

### End-points

The primary end-point was metastasis-free survival (MFS), calculated from the date of CT 1 (first chemotherapy cycle) until the first of the events of metastasis or death from any cause. Overall survival (OS) was a secondary endpoint defined as the time from CT 1 until death from any cause.

The secondary endpoints also included local recurrence, defined as the time from the date of CT 1 to local recurrence (with death considered a competing event) and the proportion of patients with progression of local disease preoperatively. Lastly, surgical margin status scored as wide, marginal and intralesional, as well as R0, R1 and R2 status, are also reported.

### Statistical analyses

Descriptive analyses were used. Treatment endpoints, as well as toxicity from CT and RT, were analysed in all 20 patients who started chemotherapy.

The survival analyses were based on follow-up data up to 2 years after the last patient enrolment (which occurred on June 30, 2014), at which time the database was locked according to the a priori analysis plan. Patients with no events were censored either at the last date of follow-up or at the predefined date of data lock (June 30, 2016).

A final survival analysis, with use of follow-up data until December 31, 2019, is also presented.

The Kaplan–Meier method was used to estimate MFS and OS [19]. For the analyses of MFS, patients who were alive and disease free were censored at the date of the last follow-up, but not later than December 31, 2019. For the analyses of OS, data for patients who were alive were censored at the date of last follow-up, but not later than December 31, 2019. Cox regression analyses to study relative risks were not performed due to the low number of patients.

The dose intensity of CT was calculated using the method reported in previous studies [11, 14, 20].

## Results

### Patients

**Fig. 2:**
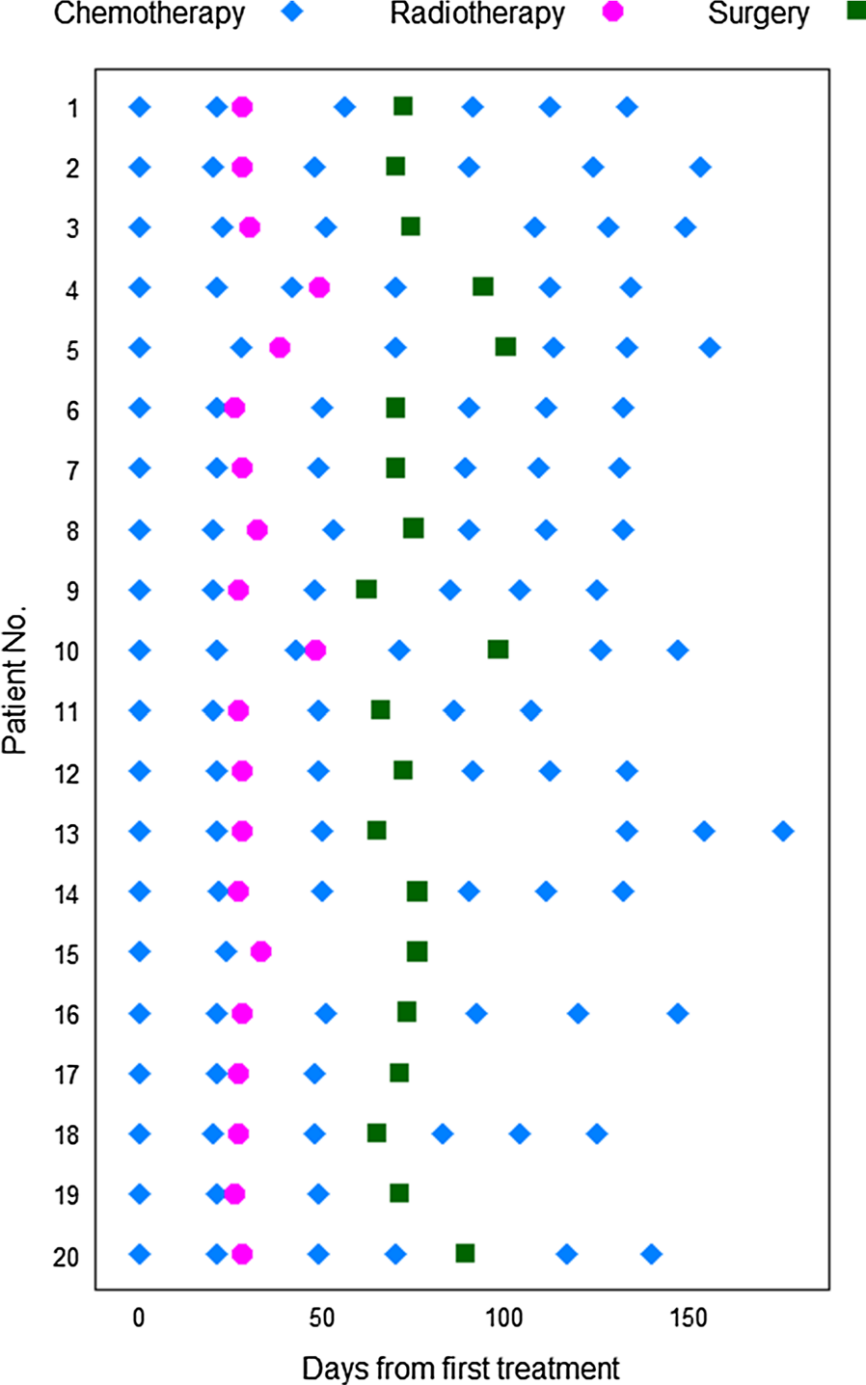
SSG XX, group B, time schedule for chemotherapy, radiotherapy and surgery

The patient demographics, tumour characteristics and histological subtypes are shown in Table 1. The timing of various treatments completed by individual patients is shown by case numbers in Fig. 2. Inclusion of patients (n = 20) in this group with preoperative treatment (group B of SSG XX) was closed in June 2014, when the planned number of patients in the postoperative and adjuvant group A (n = 160) had been fully recruited [11].

**Table 1:** Patients demographic and tumor characteristics of eligible patients

Characteristics	Numbers
Age at diagnoses (years)	
Median	59
Range	22–71
Gender	
Male	14
Female	6
Tumor site	
Lower extremity (including gluteal and groin)	12
Upper (including shoulder)	5
Trunk wall	3
Location	
Subcutanous	0
Deep	20
Tumour size (cm)	
Median	13
Range	7–17
Histopathological subtype	
Undifferentiated pleomorphic sarcoma	10
Pleomorphic liposarcoma	1
Leiomyosarcoma	1
Synovial sarcoma	6
Malignant peripheral nerve sheath tumor	1
Myxofibrosarcoma	1

Nineteen patients had limb-sparing primary surgery; only in one patient was an amputation deemed necessary (case 8). Hence, the latter patient was not evaluable for scoring of any wound complications related to RT. This patient had a synovial sarcoma (13 cm) located in the distal forearm, with close relation to vessels and nerves, but after 3 CT cycles and RT, the tumour was unchanged (MRI). He therefore underwent amputation 4 weeks later, distal to the elbow, with a 5 cm proximal margin from the tumour followed by the 3 remaining CT cycles. He developed a solitary pulmonary metastasis (45 mm) that was surgically removed 3.5 years later and is NED as of his last follow-up after 8 years.

Importantly, none of the 20 patients experienced local tumour progression preoperatively.

Resection margins were wide (R0 resection) in 13 patients, marginal (R0) in 6 patients (cases 1, 5, 9, 12, 13 and 16) and intralesional (R1) in 1 (case 2).

### Metastases-free and overall survival

**Fig. 3:**
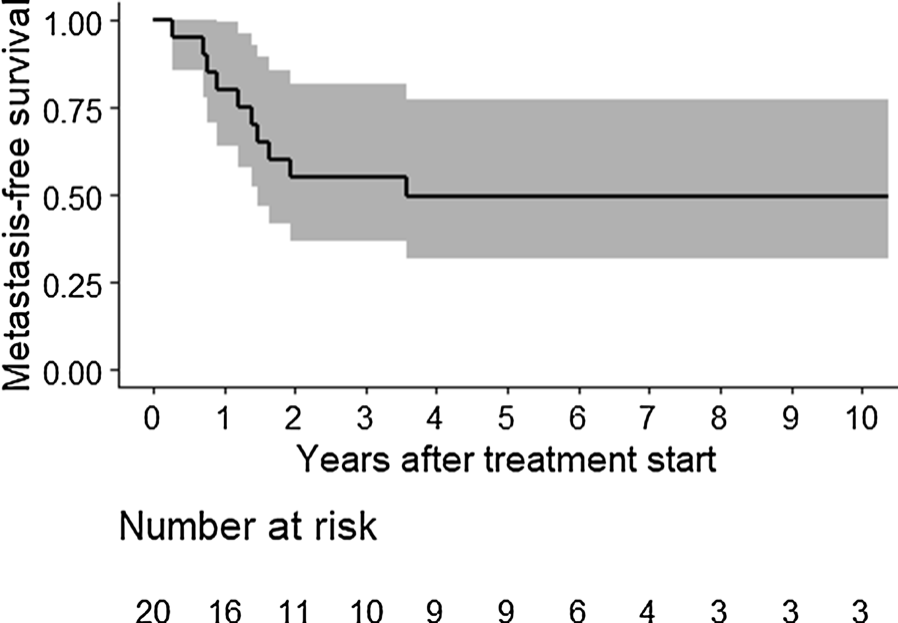
Metastases-free survival (Kaplan–Meier Curve; % with 95% CI = confidence interval) of 20 patients with high risk soft tissue sarcoma and a median follow-up 2.8 years

**Fig. 4:**
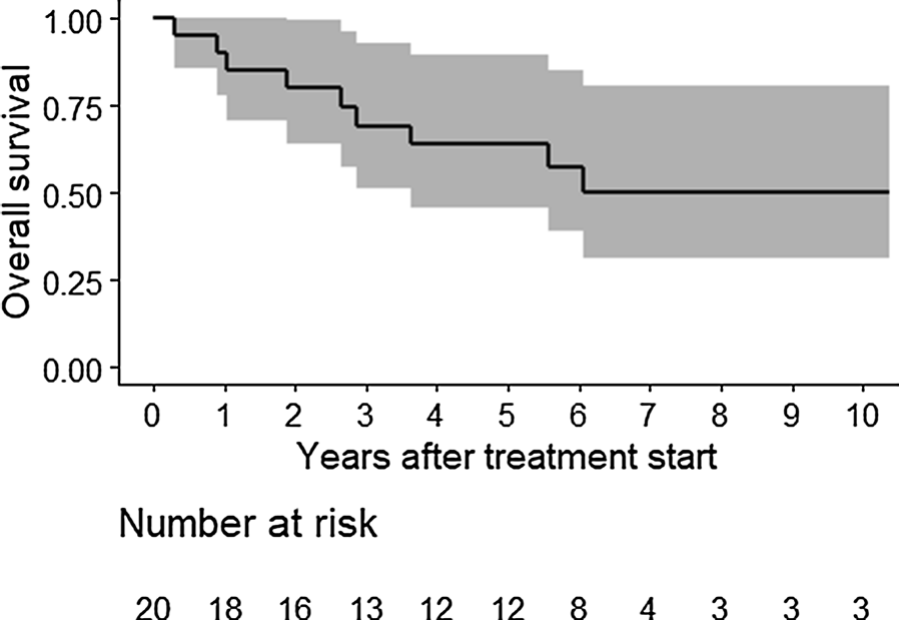
Overall survival (Kaplan–Meier Curve; % with 95% CI = confidence interval) of 20 patients with high risk soft tissue sarcoma and a median follow-up 5.4 years

The analysis according to the described statistical plan in the protocol was done based on the data collected up to a time point exactly 2 years after complete enrolment in the SSG XX trial. By that definition, the data cut-off became June 30, 2016. Later events were therefore not included in this first analysis. As stated above, we also decided to make a later analysis for group B (not done for group A) since several years had elapsed. This analysis included all data up to December 31, 2019. Survival curves for these late analyses only are shown (Figs. 3, 4).

Median follow-up of patients in the pre-planned analyses of the primary end-point MFS was 2.1 years (range 0.3–10.4). The estimated MFS rate at 5 years was 44.0% (95% CI 24.4–79.5). For overall survival (OS), the median follow-up time was 3.1 years (range 0.3–10.4). The 5-year estimated OS was 66.7% (95% CI 47.7–93.2).

As of the end of 2019, distant metastases had occurred in 10 patients (50%), 9 to the lung (cases 2, 7, 8, 9, 10, 11, 14, 15 and 16) and one to the cerebrum (case 17). Nine patients had died (45%), all from sarcoma metastases. The median follow-up of patients in this analysis of MFS was 2.8 years (range 0.3–10.4). The final estimated MFS rate at 5 years was 49.5% (95% CI 31.7–77.4) (Fig. 3). For overall survival (OS), the median follow-up time was 5.4 years (range 0.3–10.4). The final estimated 5-year OS was 64% (95% CI 45.8–89.4) (Fig. 4).

### Chemotherapy cycles and dose intensity

Sixteen patients received all the planned 6 cycles, 1 patient had 5 cycles, 2 patients 3 cycles and 1 patient had 2 cycles only (Fig. 2). Median dose intensity for doxorubicin and ifosfamide combined was only 79.0% (range 33–86).

For logistical reasons two patients had 3 cycles of CT before RT and three patients received 4 cycles before surgery (Fig. 2). One patient who had surgery after only 2 cycles of CT and RT did not have more CT due to a non-healing surgical wound complication (case 15). She later died of metastases. Two patients who had 3 preoperative cycles did not receive postoperative treatment. In one this was due to cerebral metastases diagnosed shortly after surgery with rapid clinical deterioration and death (case 17), and in the other due to refusal of postoperative CT (case 19).

Only 3 patients had their first postoperative cycle as scheduled within 15 days postoperatively (cases 5, 8 and 14); 10 patients had this cycle between day 15 and 23, 3 patients between day 28 and 34 (cases 3, 10 and 20) and 1 patient at day 68 (case 13).

### Chemotherapy toxicity

The CT toxicity was recorded according to Common Terminology for Adverse Events (CTCAE), version 3.0. In general, CT was well tolerated and no worse than in our earlier described larger SSG XX group of patients who only underwent postoperative CT and RT [11]. Table 2 presents all toxicities reported in more than one patient and numbers that had grade 3–4 toxicity. Neutropenia, without fever and thrombocytopenia, was the most common side effect. One case of pulmonary embolism grade 4 was reported as the most serious toxicity. No fatal toxicities occurred.

**Table 2:** Toxicity according to CTCAE score

Toxicity	All grades^a^	Grade 3–4^b^
	Number of patients	Number of patients
Neutropenia without fever	17	12
Neutropenia with fever	3	3
Thrombocytopenia	16	6
Anaemia	3	3
Wound infection	2	2
Other infections, normal ANC	3	0
Haematuria	3	3
ALAT increased	6	0
Creatinine increased	2	0
Fever, unspecified	2	0
Pulmonary embolism	1	1

^a^All grades of toxicities occurring in > 1 patient or any grade 3–4

^b^Number of patients with CTCAE toxicities grade 3–4

Long-term cardiac and renal toxicities were measured by left ventricular ejection fraction (LVEF) and glomerular filtration rate (GFR), respectively, at several time points during and after the treatment. The last measurements were scheduled, according to the SSG XX protocol, at 10 years after end of treatment. The majority of the patients did not show any late cardiac or renal effects, but several data points were missing (i.e. the investigations were not performed). Some patients also had very few, if any, measurements because of rapid disease progression dismissing the study-related follow-up. Among the 15 and 16 patients who had at least one measurement of cardiac and renal toxicity, respectively, four patients showed a grade 1 cardiac toxicity and just one had grade 1 renal toxicity.

### Radiotherapy and toxicity

Eighteen patients received the scheduled RT to 36 Gy preoperatively after 2 cycles without any delays (Fig. 2). Acute RT toxicity was recorded at each chemotherapy cycle until 6 weeks after last cycle. The RTOG Acute Radiation Toxicity Scoring was used [21].

Maximum skin toxicity due to RT during the treatment period was grade 4 in 1 patient (5%), grade 3 in 4 patients (20%), grade 2 in 6 patients (30%) and grade 1 in 5 patients (25%). Scoring 0 was reported for 4 patients. In 2 patients only, toxicity resulted in a delay of CT. Notably, very few patients had late radiotoxicity.

None of the patients developed a second cancer.

### Local recurrence rate

Three patients developed a local recurrence (LR). This related to one patient with wide margin by primary surgery of a tumour (12 cm, UPS) in lower extremity that developed LR 5.5 years after primary surgery (5.8 years after cycle 1) and was salvaged by an uncomplicated re-operation. The patient was NED at follow-up 3 months later (case 3). Furthermore, two patients, both having 10 cm tumours (synovial sarcoma) in the lower extremities had LR 18 months after primary surgery (20 and 21 months after cycle 1, respectively). The margins at primary surgery were wide (R0) and intralesional (R1), respectively. Interestingly, both these patients experienced LR after first being diagnoses with pulmonary metastases (11 months and 4 months, respectively). Both died later of metastases (cases 2 and 11).

### Surgical complications

No wound healing complications were observed in 8 patients. Delayed wound healing in ranges of 2 to 4 months occurred in 7 patients (cases 9, 11, 12, 13, 14, 19 and 20) and lasting as long as 6 months in 1 patient (case 5). These patients all had early postoperative infections that were treated with antibiotics and wound revisions followed by vacuum pump treatment. One patient needed a skin transplant and healing occurred at 6 months (case 5). Three patients (cases 6, 15 and 17) had persistent wounds that did not heal, and two of them died early due to metastatic disease (cases 15 and 17). The third patient (case 6) was NED at last follow-up in 2019 but with a still unresolved non-healing wound after multiple revisions and hyperbaric oxygen treatment.

## Discussion

This study explored the possible benefit of combining neoadjuvant CT and preoperative, accelerated RT in a defined cohort of patients with high-grade STS, where an adequate surgical margin of primary surgery could not a priori be achieved (SSG XX, group B). Interestingly, the surgical margins were in fact intralesional (R1) in only one patient. This regimen may thus be warranted for large extremity localised STS and proximally located tumours in the lower extremity/groin, when preservation of function is the goal and the tumour is located near critical structures, such as larger vessels and nerves. The local control rate was deemed satisfactory, as only 3 of the 20 patients experienced a local recurrence and in two this occurred after diagnosis of metastatic disease. Nine patients developed pulmonary metastases. Only one patient underwent a complete metastasectomy, with NED observed after 8 years. The 5-year estimated MFS and OS rates were 49.5% and 64.0%, respectively.

The Italian and Spanish Sarcoma Groups (ISG/GEIS) administered neoadjuvant and adjuvant CT with doxorubicin and ifosfamide to similar patients. They concluded that CT may be omitted after 3 preoperative cycles, based on their study comparing treatment with 3 preoperative cycles only with that of addition of two further postoperative cycles [9]. The 10-year overall survival for both treatment arms was about 60% [22]. Interestingly, our survival data seems to be in accordance with the ISG/GEIS trial. In the same study, preoperative RT (44–50 Gy) was given to 169 patients (from a total of 303 patients) and wound complications occurred in 13.5% [3]. Gronchi et al. showed in a recent publication that disease free and overall survival by histiotype-tailored neoadjuvant chemotherapy was not superior to anthracycline and ifosfamide in high-grade STS [23]. Their conclusion was that doxorubicin and ifosfamide should remain the regimen to choose whenever neoadjuvant chemotherapy (or adjuvant) is used.

As already mentioned, the addition of preoperative RT to CT is adopted at many centres as part of a multimodal treatment plan for patients with primary STS [1, 2, 10, 24]. We did not include specific assessment of response into the protocol, neither by radiology nor pathology [18]. It was, however, mandatory to repeat MRI before surgery (after 3 preoperative cycles + radiotherapy), but in the clinical report forms only preoperative tumour progression was registered. Interestingly, no patients had preoperative tumour progression after a composite radiological/clinical evaluation at the local hospital. Furthermore R0 resection margin was obtained in 19 patients (of 20) which for all practical reasons is an important result, since all eligible patients in SSG XX, group B should have an obvious risk for intralesional surgery. In the ISG/GEIS study reported above, some cases had positive microscopic margins, despite preoperative CT and RT, but this was not associated with an increased risk of distant spread or local recurrence [25].

Several studies have demonstrated no difference in the rates of local tumour control between pre-and postoperative RT [26–29]. The optimal timing of RT is debated and depends on factors such as tumour location and patient characteristics [30]. In patients where no preoperative RT has been given, the current consensus is that postoperative RT is indicated in all high-grade STS following marginal and intralesional margin surgery, as well as after wide and marginal margin surgery in deep-seated, high-grade tumours [4].

The value of preoperative RT for improving survival of patients with STS remains unproven [27]. O`Sullivan et al. [26] randomised 94 patients to preoperative (50 Gy, 25 fractions) or postoperative (66 Gy, 33 fractions) RT and showed a slightly better overall survival in the preoperative group after a median follow-up time of 3.3 years. However, this benefit was lost after 5 years of follow-up (recurrence-free survival: 58% versus 59%) [31]. The timing of RT did not affect local control, but more patients had wound complications in the preoperative RT group than in the postoperative group [26]. Other researchers have also reported increased risk of wound complications [1, 32]. Nevertheless, preoperative RT is preferred by many due to its more favourable long-term risk profile, with less fibrosis, joint stiffness and oedema [31, 33, 34]. The temporary nature of wound complications also motivates preoperative RT [30].

In the recently reported phase 3 study by ISG/GEIS, where preoperative RT was combined with doxorubicin and ifosfamide (3 cycles), the incidence of wound complications was 17% whereas it was 10% with neoadjuvant CT, alone or combined with postoperative RT [3]. In our present study, 11 of 20 patients had wound complications, but these were mostly of short duration. Possibly, our 3 additional cycles postoperatively might have increased the toxicity. One factor to be kept in mind is that the acute toxicity profile associated with preoperative RT may be mitigated by experienced surgeons using techniques such as free and pedicle flaps for high-risk locations. Therefore, these patients should be referred to specialised centres [25, 35]. All patients in our study were treated by experienced sarcoma surgeons.

For preoperative RT a dose of 50 Gy in 1.8–2 Gy in once-daily fractions over 5–6 weeks is the usual delivery schedule [4, 35]. Accelerated RT (36 Gy, 20 fractions of 1.8 Gy per fraction, twice daily, 5 days per week) is equivalent to a total dose of about 50 Gy [18]. The accelerated RT interposed between CT cycles was used in SSG`s former adjuvant protocol, SSG XIII [14] and subsequently in SSG XX where higher CT doses were given (group A) [11]. Acceptable treatment-related morbidity was demonstrated [11, 14]. In the current study we evaluated accelerated RT in a preoperative setting in highly selected patients (group B). Accelerated radiotherapy interposed between CT cycles may allow the maintenance of a high overall dose intensity of the CT given as evident for both group A [11] and group B.

In a retrospective study, 89 patients with localised high risk STS had six courses of doxorubicin (50 mg/m^2^) and ifosfamide (5 g/m^2^) and hyperfractionated radiotherapy (1.5 Gy twice daily/ 42–60 Gy), given as preoperative treatment in 45 patients and as postoperative treatment in 44 patients [36]. In that study, the treatment-related complication rate was moderate, with a 5-year MFS of 48% and a local control rate of 81%. The doses of CT and the dose per fraction of RT were lower than in our SSG XX study [11]. In the retrospective study reported [36] and in the previous SSG XIII study [14], it was shown that a low CT dose intensity had a negative impact on both metastasis-free and overall survival. For inadequate surgical margins, the hyperfractionated RT was given as a split-course procedure [36]. Their relatively high local recurrence rate might underscore the findings of Jebsen et al. [14], who showed that split-course RT to a greater total radiation dose could not compensate for poor surgical margins.

More recently, Spalec et al. showed in a prospective study of 30 patients with borderline resectable STS that radiotherapy with 5 fractions of 5 Gy combined with preoperative chemotherapy was feasible [37]. R0 resection margin was obtained in 15 of the 23 patients and R1 in 7 patients who had limb-sparing surgery. By pathological evaluation of the removed tumours < 50% stainable cells was found in 14 patients. Good early tolerance of the treatment was reported.

The non-randomised design and the low sample number in our study limit the interpretation of the results. The study strengths were the prospective design and the strict high risk inclusion criteria. We believe that our findings may add valuable information for clinical teams treating this particular and challenging group of STS patients.

## Conclusions

A low local recurrence rate (3 of 20 patients) with an acceptable 5-year estimated MFS (49.5%) and OS (64.0%) rate were demonstrated in a very high risk group of STS patients (SSG XX, group B). Only one patient required an amputation. A combined preoperative CT and RT approach resulted in almost all operations rendering adequate surgical margins. Wound healing problems occurred in several patients but were mostly of a temporary nature. From these results we recommend preoperative chemotherapy and radiotherapy to be considered for selected high risk patients after careful evaluation by a multidisciplinary team. Referral of STS patients to specialised centres is always recommended, and this is particularly important in high-grade locally advanced cases.

## Data Availability

The presented data are available from the corresponding author.

## Abbreviations

MFS: Metastasis-free survival
OS: Overall survival
LR: Local recurrence
RT: Radiotherapy
CT: Chemotherapy
MRI: Magnetic resonance imaging
SSG: Scandinavian Sarcoma Group
STS: Soft tissue sarcoma
WHO: World Health Organization
NED: No evidence of disease
